# Targeted Expression of Suicide Gene by Tissue-Specific Promoter and MicroRNA Regulation for Cancer Gene Therapy

**DOI:** 10.1371/journal.pone.0083398

**Published:** 2013-12-31

**Authors:** Ravikanth Danda, Gopinath Krishnan, Kalaivani Ganapathy, Uma Maheswari Krishnan, Khetan Vikas, Sailaja Elchuri, Nivedita Chatterjee, Subramanian Krishnakumar

**Affiliations:** 1 Department of Ocular Pathology, Vision Research Foundation, Sankara Nethralaya, Chennai, India; 2 Centre for Nanotechnology and Advanced Biomaterials, Shanmugha Arts, Science, Technology and Research Academy University, Tanjore, India; 3 Departments of Ocular Oncology and Vitreoretina, Medical Research Foundation, Sankara Nethralaya, Chennai, India; University of Missouri-Columbia, United States of America

## Abstract

In order to realise the full potential of cancer suicide gene therapy that allows the precise expression of suicide gene in cancer cells, we used a tissue specific Epithelial cell adhesion molecule (EpCAM) promoter (EGP-2) that directs transgene Herpes simplex virus–thymidine kinase (HSV-TK) expression preferentially in EpCAM over expressing cancer cells. EpCAM levels are considerably higher in retinoblastoma (RB), a childhood eye cancer with limited expression in normal cells. Use of miRNA regulation, adjacent to the use of the tissue-specific promoter, would provide the second layer of control to the transgene expression only in the tumor cells while sparing the normal cells. To test this hypothesis we cloned let-7b miRNA targets in the 3’UTR region of HSV-TK suicide gene driven by EpCAM promoter because let-7 family miRNAs, including let-7b, were found to be down regulated in the RB tumors and cell lines. We used EpCAM over expressing and let-7 down regulated RB cell lines Y79, WERI-Rb1 (EpCAM^ +ve^/let-7b^down-regulated^), EpCAM down regulated, let-7 over expressing normal retinal Müller glial cell line MIO-M1(EpCAM ^−ve^/let-7b^up-regulated^), and EpCAM up regulated, let-7b up-regulated normal thyroid cell line N-Thy-Ori-3.1(EpCAM^ +ve^/let-7b^up-regulated^) in the study. The cell proliferation was measured by MTT assay, apoptosis was measured by probing cleaved Caspase3, EpCAM and TK expression were quantified by Western blot. Our results showed that the EGP2-promoter HSV-TK (EGP2-TK) construct with 2 or 4 copies of let-7b miRNA targets expressed TK gene only in Y79, WERI-Rb-1, while the TK gene did not express in MIO-M1. In summary, we have developed a tissue-specific, miRNA-regulated dual control vector, which selectively expresses the suicide gene in EpCAM over expressing cells.

## Introduction

Conventional treatments like chemotherapy, radio therapy and surgery are the most efficient means to treat cancer patients [1]. Based on the disease stages, current treatment methods for retinoblastoma (RB) the most frequent neoplasm of the eye in childhood, include intensive chemotherapy, Radiation, consolidation with autologous hematopoietic stem cell rescue and surgical resection. However, patients with vitreous seeds, sub retinal seeds, and bilateral advanced multifocal diseases are a major challenge with the current treatment options [2], [3]. Recent discovery of cancer stem cells, a subset of cancer cells with stem cell-like properties which is in part, responsible for tumor growth with different properties compared to differentiated tumor cells are increasing the complexities of cancer treatment [1]. Therefore, new treatment methods are needed to fight against cancer. Among various approaches, suicide gene therapy could be a promising alternative strategy since the suicide gene expression can be regulated to a particular tissue [4], [5]. Suicide gene therapy involves the intracellular delivery of a gene coding for an enzyme that transforms a prodrug into a cytotoxic product [6], [7]. The most commonly used suicide gene is the herpes simplex virus type I thymidine kinase (HSV-TK). Various studies had used HSV-TK suicide gene therapy to treat RB and other cancers [8], [9], [10], [11], [12], [13], [14]. Non specific expression of suicide gene in normal cells is a serious limitation in the existing suicide gene strategies for cancer therapy.

In order to efficiently control the transgene expression only in target cells, various studies have used different tissue specific promoters [15], [16], [17], [18], [19], [20]. One such promoter is epithelial glycoprotein-2/EpCAM/17-1A (EGP2) promoter which selectively kills the EpCAM over expressing cells in many cancers by restricted expression of TK (thymidine kinase) followed by Ganciclovir (GCV) treatment [21], [22]. EpCAM/CD326 is a type I trans membrane glycoprotein which is expressed in apical membrane of cancer cells and shows baso-lateral expression in normal epithelial cells. It has been reported to be specifically expressed in epithelial tissue, and over expressed in majority of human epithelial carcinomas, including colorectal, breast, prostate, head and neck, hepatic carcinomas and retinoblastoma [23], [24]. Controlled gene expression in the targeted tissues is crucial for the gene therapy particularly in the context of cancer suicide gene therapy. Even though tissue specific promoter driven suicide gene therapy showed promising results, leaky expression of the tissue specific promoters in non targeted cells has been reported [21]. Therefore, alternative strategies are essential in addition to the tissue specific promoter regulation of the suicide gene therapy.

MicroRNAs (miRNAs) are a class of small, non-coding RNAs (>1000 in mammalian cells) that regulate various cellular functions ranging from cell division, signal transduction and metabolism. The posttranscriptional regulation of gene expression via miRNA-mediated RNA interference (RNAi) is well known [25]. miRNAs are known to block mRNA translation or reduce mRNA stability after perfect binding of the guide strand to the miRNA-recognition elements (MREs) within the 3^l^untranslated region (UTR) of the target genes [26], [27], [28], [29]. let-7 is a class of miRNAs which are involved in cell regulation and tumor suppression. Earlier studies on prostate, lung and breast cancers, showed down regulation of the let-7 family miRNAs [30], [31]. Recently, Glial fibrillary acidic protein (GFAP) promoter driven tissue specific gene expression vectors along with the target sequences for the deregulated miRNA (mir122a, mir31, mir127, mir143) were introduced into the 3’UTR region of the transgene [32], [33], [34], [35], [36]. This approach had allowed the transgene expression only in targeted glioblastoma cancer cells without any expression in normal astrocytes cells of the same lineage [34].

In this study, we constructed a mammalian cell expression vector, which harbours EpCAM promoter (EGP2) driven suicide gene HSV-TK regulated by the expression of the deregulated miRNA levels. The aim is to express the suicide gene only in EpCAM^+ve^ cells with very minimal or no expression in normal cells of the same lineage. The let-7b miRNA is under expressed in Y79, WERI-Rb-1 cancer cell lines. We made EGP2-TK construct with 2 and 4 copies of let-7b miRNA target sequences. The expression of TK gene is restricted to cancer cell lines, such as Y79, WERI-Rb-1, MDA-MB-453, and MCF-7 whereas TK gene expression is minimal in MIO-M1 cell line. We demonstrated for the first time the use of EpCAM promoter driven miRNA regulated TK Suicide gene in EpCAM positive and let-7 down regulated cells. This strategy offers a two tier level control of the transgene that may lead to a selective transgene expression in the cancer cells without any expression in the normal cells.

## Materials and Methods

### Ethics statement

Retinoblastoma tumors samples were collected from enucleated eye balls as part of therapy and utilized for research purpose, a written general consent was obtained from the parents/guardians of the patient undergoing enucleation. The study was conducted after obtaining the approval from the Ethics Sub-Committee (Institutional Review Board) of Sankara Nethralaya eye hospital [Ethical clearance no: 147(A)-2009-P].

### Tumor samples and pathological details

For this study 10 fresh retinoblastoma tumors were used, Clinocopathological details for the tumors were based on international retinoblastoma staging working group (IRSWG) [37], [38] and were given in table S1. Pooled adult retina from 50–55(n = 3) years old were used as a control and were obtained from cadaveric eyes donated to C.U Shah Eye Bank, Sankara Nethralaya.

### Cell culture

The retinoblastoma cell line Y79, WERI-Rb1, and Breast cancer cell lines MDA-MB-453, MCF-7 were obtained from RIKEN Bio Resource Centre (Japan). Human Retinal Müller glial cell line MIO-M1 derived from the neural retina was gifted from G.A. Limb, UCL Institute of Ophthalmology, London, England [39]. Human thyroid follicular epithelial cell line Nthy-ori 3-1 was gifted from Dr.Omer Ugur, Hacettepe Institute of Oncology [40], [41]. MIO-M1, MDA-MB-453, MCF-7 were cultured in Dulbecco’s modification of Eagle’s media (DMEM) containing glutamine, supplemented with 10% heat-inactivated fetal bovine serum (FBS), penicillin (100 IU/ml) and streptomycin (100 IU/ml). Nthy-ori 3-1 and Y79, WERI-Rb1 were cultured in RPMI1640 supplemented with 10% heat-inactivated fetal calf serum, 2 mM L-glutamine, 1 mM sodium pyruvate, and 4.5% dextrose and grown in suspension at 37°C in a 5% CO_2_-humidified incubator.

### *In vitro* transgene expression analysis

(EGP2-TK) was kindly provided by Dr. MG Rots, Department of Therapeutic Gene Modulation, University Centre for Pharmacy, University of Groningen, The Netherlands. The pUT649 expression vector containing a Zeocin resistant gene and carrying the HSV-TK gene under the control of human cytomegalovirus promoter (CMV-TK) [generated by Professor G.Tiraby (University of Toulouse), Cayla, France] were transiently transfected into Y79, WERI-Rb1, Nthy-ori 3-1, MIO-M1 using Lipofectamine TM 2000 transfection reagent (Invitrogen, USA) according to the manufacturer’s instructions.

### Cloning of miRNA targets in the EGP2-TK plasmid

To construct the plasmids with let-7 miRNA target sequences, EGP2-TK was used as a backbone containing HSV-TK in the downstream of EGP2 promoter. For constructing the miRNA targets, oligonucleotides were designed with let-7b targets (perfect complement sequence of let-7b miRNA was taken from miRBase accession no-MIMAT0000063) followed by *KpnI* which are designated as T1A (having targets perfect reverse complementary to the let-7b miRNA) and T1B (perfectly complementary to T1A) were annealed and ligated between *NotI* and *BstBI* restriction sites resulting in EGP2-TK-2T having 2 copies of the let-7b targets. The *KpnI*/*BstBI* fragment was digested with respective enzymes and ligated with the oligonucleotides having two more copies of let-7b target sequences. Which is designated as T2A (having targets perfect complementary to let-7b miRNA) and T2B (perfectly complementary to T2A) respectively. These were annealed and ligated resulting in EGP2-TK-4T having 4 copies of let-7b target sequences. The control targets C1A, C1B and C2A, C2B were generated by taking the perfect reverse complementary of the let-7b miRNA target. Throughout the manuscript EGP2-TK with 2 copies of let-7b targets will be mentioned as EGP2-TK-2T and 4 copies of let-7b targets as EGP2-TK-4T. In the same way EGP2-TK with 2 copies of let-7b control sequence will be referred as EGP2-TK-2C and 4 copies of control sequence will be denoted as EGP-TK-4C. The plasmid constructs for luciferase (luc) reporter assay were generated by replacing the HSV-TK gene with the *Guassia luciferase* gene. The luciferase constructs were named as mentioned above replacing TK with luc. Table 1 lists the oligonucleotides used in the study.

**Table 1:** Oligonucleotides used in this study.

**let-7b-T1A**	5^|^ GGCCGCAACCACACAACCTACTACCTCACAGC AACCACACAACCTACTACCTCAGGTACCGCGCATGCATT 3^|^
**let-7b-T1B**	5^|^ CGAATGCATGCGCGGTACCTGAGGTAGTAGGTTGTGT GGTTGCTGTGAGGTAGTAGGTTGTGTGGTTGC 3^|^
**let-7b-T2A**	5^|^ CAACCACACAACCTACTACCTCA TATGAACCACACAACCTACTACCTCAATT 3^|^
**let-7b-T2B**	5^|^CGAATTGAGGTAGTAGGTTGTGTGGTT CATATGAGGTAGTAGGTTGTGTGGTTGGTAC 3^|^
**let-7b-C1A**	5^|^GGCCGCACTCCATCATCCAACACACCAACAGCACTCCATCATC CAACACACCAAGGTACCGCGCATGCATT 3^|^
**let-7b-C1B**	5^|^CGAATGCATGCGCGGTACCTTGGTGTGTTGGATGATGGAGTGC TGTTGGTGTGTTGGATGATGGAGTGC 3^|^
**let-7b-C2A**	5^|^CACTCCATCATCCAACACACCAACATATGACTCCATCAT CCAACACACCAAATT 3
**let-7b-C2B**	5^|^CGAATTTGGTGTGTTGGATGATGGAGTCATATGTTGGTGTGTT GGATGATGGAGTGGTAC 3^|^

### miRNA expression analysis

The TaqMan® MicroRNA Reverse Transcription Kit and the TaqMan® Universal PCR Master Mix without Amperase® UNG from Applied BioSystems (CA, USA) was used for the detection and quantification of mature miRNA. Quantification was done according to the manufacturer’s protocol using 1 µg of total RNA sample. We used RNU6 miRNA (assay ID, 001093) as a control. TheTaqMan® MicroRNA (Applied Biosystems) individual assays were used for the following miRNA estimations: hsa-let-7a (assay ID, 000377), hsa-let-7b (assay ID, 2619), hsa-let-7c (assay ID, 000379), hsa-let-7d (assay ID, 002283), hsa-let-7e (assay ID, 2406), hsa-let-7f (assay ID, 000382), and hsa-let-7g (assay ID, 0002282) and hsa-let-7i (assay ID, 002221). Data were normalized using Ct values for the house-keeping gene U6 in each sample. To calculate relative amounts of miRNA, the average Ct value of the U6 RNA was subtracted from that of the target miRNA to obtain the change in Ct value. The fold change in gene expression was then determined as log2 relative units. The PCR products were detected with an ABI PRISM 7500 sequence detection system and analysed with the ABI PRISM 7500 SDS software (Applied Biosystems).

### Reporter gene assay

We used luciferase reporter gene assay for promoter strength analysis and studying the miRNA regulation on the transgene expression. pGluc plasmid containing the CMV promoter driven -luciferase construct was purchased from (New England Biolabs, MA, USA). The EGP2 promoter was cloned in front of a luciferase gene (EGP2-luc) replacing the CMV promoter. The 2 and 4 copies of let-7b target sequences were cloned at the 3’UTR region of the construct (EGP2-luc). The transfection was done transiently with the following plasmids CMV-luc, EGP2-luc, EGP2-luc-2T, EGP2-luc-4T, EGP2-luc-2C and EGP2-luc-4C. After 48 h of transfection with luciferase construct, cells were washed with PBS and grown in fresh medium for an additional 24 hours. The cells were harvested and analysed for luciferase and β-galactosidase activities in accordance with the manufacturer’s protocols (New England Biolabs).

### Western blot analysis

In order to analyse the expression of TK and EpCAM proteins, total cell extracts were prepared by lysing the cells in the lysis buffer [10 mM Tris-HCl pH 7.2, 2% SDS, 10 mM dithiothreitol, 1% protease and phosphatase inhibitors cocktail (Sigma Aldrich, MO, USA)]. The protein concentration was estimated by Lowry’s method. The cell lysates were mixed with 5X Laemmli loading buffer and boiled for 5 min. Equal amounts (50μg) of protein were subjected to electrophoresis on a 12% SDS-polyacrylamide gel and electro-blotted to nitrocellulose membrane. The membrane blots were blocked for 1 h with 5% skimmed milk in TBS containing 0.1% Tween-20 and then incubated overnight at 4°C with primary antibody. The HSV-TK (provided by Dr. William C. Summers, Yale University) and EpCAM (Cell signalling, MA, USA) antibodies were used at the dilution of 1∶100 and 1∶150, respectively. The blots were washed and incubated with appropriate horseradish peroxidase-conjugated secondary antibodies (anti-rabbit 1∶5000; anti-mouse 1∶2000 (Cell signalling) for 3 hours at room temperature and visualized with Tetramethyl benzidine/Hydrogen peroxide (TMB/H_2_O_2_, Bangalore Genei, India) reagent according to the manufacturer’s instructions. Membranes were stripped using 100 mM glycine, pH 2, for 40 min, blocked, and re-probed for β-actin (Sigma). The blots were scanned with a UVP BioDoc-IT imaging system (CA, USA) and Densitometric analysis of digitized images was performed with ImageJ software (NIH). The intensity for each band was normalized to the intensity of the corresponding β-actin band after the background normalization.

### Measurement of sensitivity to Ganciclovir (GCV)

The Y79, WERI-Rb1, Nthy-ori 3-1, MIO-M1cells were seeded in a 96 well plate 24 h prior to the transfection at a density of 10,000 cells per well to determine the cell viability. The transfection was done transiently with plasmids containing CMV-TK, EGP2-TK, EGP2-TK-2T, EGP2-TK-4T, EGP2-TK-2C, EGP2-TK-4C and incubated for 48 h. The cells were treated with various concentrations (1, 10, 50,100 µM) of GCV for 24 h. The sensitivity to GCV was evaluated using the colorimetric MTT assay. A multi-well scanner (BioTek, VT, USA) was used to measure the absorbance at 570 nm wavelength. The non-transfected untreated controls were assigned a value of 100%. Cell survival was calculated by the equation: Test OD/Control OD X 100%.

### Immunofluorscence

The following transfected cell lines MIO-M1, Nthy-ori-3-1, WERI-Rb1, Y79 cells were washed with 1×PBS and fixed with 4% paraformaldehyde for 10 min at room temperature and then permeabilised with 0.25% Triton X-100 for 5 min. The cells were blocked with 5% fetal bovine serum for 30 min,incubated with anti- cleaved caspase 3 (Cell signaling) and anti-Bcl2 antibody (cell signaling) for 3 hours, then with FITC (green) and TRITC (red) conjugated secondary antibodies (Jackson Immunoresearch, PA, USA) for 2 h. The cells staining were visualized using a Zesis Axio vison inverted system microscope with 10X objective. To ensure specificity of staining, images were obtained using settings at which no fluorescence was detectable in negative controls with secondary antibodies alone (data not shown).

### Statistical analysis

Data are expressed as mean ±SD of 3 experiments with each experiment done in triplicates. Statistical significance was calculated with Student’s t test and a p value ≤ 0.05 was considered significant.

## Results

### Restricted TK expression by EGP2 promoter

**Figure 1:**
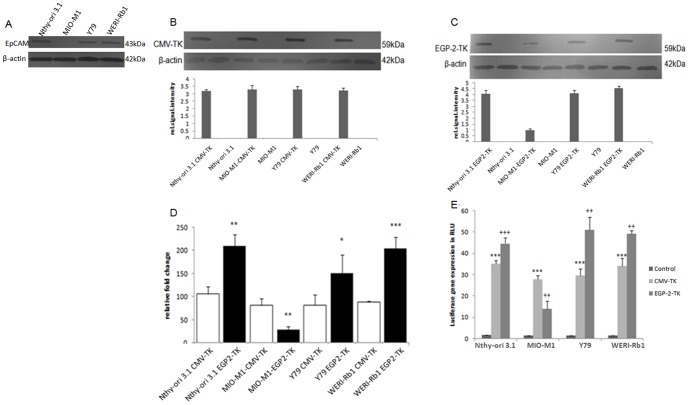
*In vitro* promoter strength analysis by expressional studies of transgene driven by both EGP2 and CMV promoter in cancerous and normal cell lines. EpCAM expression was evaluated by western blot analysis and β-actin was used as a loading control (A). The transgene expression driven by EGP2 and CMV promoter was evaluated by transient transfection of CMV-TK, EGP2-TK, CMV-luc, and EGP2-luc. After 2 days of transfection TK protein expression driven by CMV promoter was evaluated by western blot analysis and the results were normalized against the β-actin (B). TK protein expression driven by EGP2 promoter was evaluated by western blot analysis and results were normalized against the β-actin (C). Protein quantification was quantified by using ImageJ software and protein expression was expressed in relative signal intensity values. TK mRNA levels were quantified by Real-time PCR, the mRNA levels were normalized against the GAPDH and were expressed in relative fold change units (D). Luciferase expression analysis was performed by quantifying the secreted Gaussia luciferase. The results were normalized against un-transfected cells and were expressed in relative light units (E). Normalized levels of luciferase expression as percentage shown as mean ± S.D (n = 3). *p<0.05, **p<0.01, ***p<0.001 vs. Control, +p<0.05, ++p<0.01, +++p<0.001 vs. CMV-luc.

We analysed the selective expression of the transgene by EGP2 promoter in Y79, WERI-Rb1, Nthy-ori 3-1 cell lines. Western blot analysis showed EpCAM protein expression in Y79, WERI-Rb1, Nthy-ori 3-1 cells and its expression was not seen in MIO-MI cell line (Figure1A). Therefore, the Y79, WERI-Rb1, Nthy-ori 3-1cells were considered as EpCAM positive (EpCAM^+ve^) and MIO-MI as EpCAM negative (EpCAM^−ve^) for the EpCAM protein expression. To evaluate the EGP-2 promoter for its capacity to regulate HSV-TK expression in the EpCAM expressing cells, we transiently transfected the cells with EGP2 promoter driven HSV-TK construct (EGP2-TK). The CMV-promoter-driven HSV-TK (CMV-TK) construct was used as a positive control. In CMV-TK transiently transfected cells, TK protein was expressed in all the cell lines (Nthy-ori 3-1, Y79, Weri-Rb1 and MIO-M1) (Figure 1B). In EGP2-TK transiently transfected cells, TK expression was seen in Nthy-ori 3-1, Y79 and WERI-Rb1 cells. Furthermore, the TK protein expression was low in MIO-M1 cells (Figure 1C). This could be due to the leakiness of the EGP2 promoter as reported previously in other promoters [34], [35]. In real time PCR experiments the CMV promoter driven TK gene showed consistent expression in all the 4 cell lines (Figure 1D). EGP2-TK transfected Nthy-ori 3-1, Y79 and WERI-Rb1 cell lines showed significant higher TK expression compared to the CMV-TK transfected cell lines. However, EpCAM promoter significantly reduced TK gene expression in the MIO-MI cell line (Figure 1D). Reporter gene assay showed that CMV-luc transfection exhibited a significant higher gene expression compared to the untransfected cells. The EGP2-luc transfection into the Nthy-ori 3-1, Y79, and WERI-Rb1 cell lines showed significant higher luciferase expression compared to the CMV-luc transfected cells except in MIO-M1 where the luciferase expression is significantly lower in EGP2-luc transfection (Figure 1E). These results show that EpCAM promoter is selectively activated in EpCAM positive cell lines Y79, WERI-Rb1, Nthy-ori 3-1, and not in EpCAM negative cell line MIO-M1.

### EGP-2 promoter controls selective cell killing by restricted expression of TK

**Figure 2:**
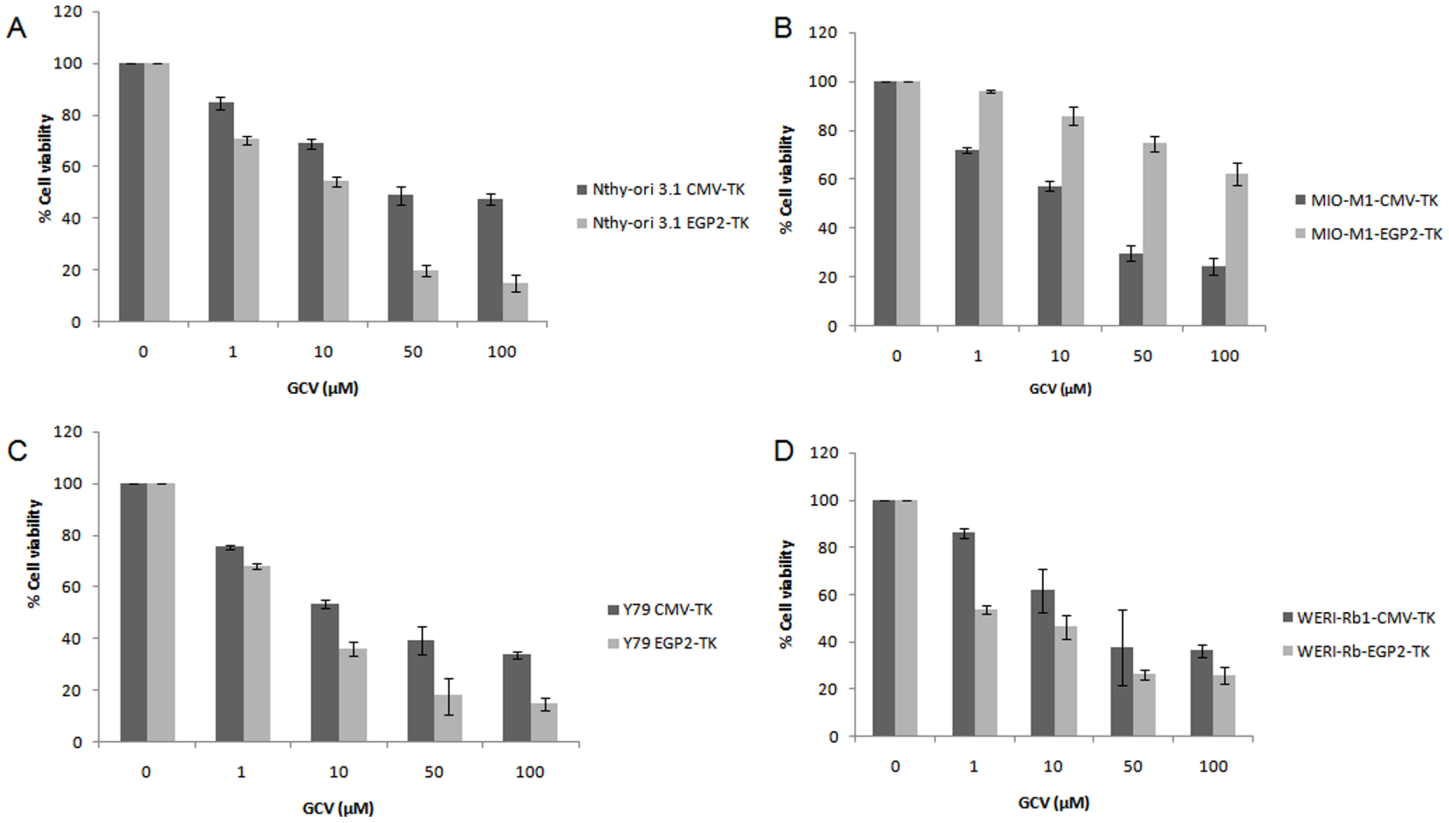
GCV sensitivity to TK transfected cells. Cell viability assay by MTT was evaluated by transfecting the EGP2-TK and CMV-TK plasmids into the cell lines. Nthy-ori-3-1 (A), MIO-M1 (B), Y79 (C), WERI-Rb1 (D). After 48 hrs of transfection, followed by GCV treatment at different concentrations (1, 10, 50, 100 µM) for 24 hrs. The results were normalized against un-transfected cells and were expressed in percentage of cell viability. Cell viability was shown as percentage mean ± S.D (n = 3).

In order to evaluate the functional aspects of the TK gene expression, different concentrations (1, 10, 50, 100 µM) of GCV treatment was done for 24 hours on CMV-TK or EGP2-TK transiently transfected cell lines such as Y79, WERI-Rb1, Nthy-ori 3-1, MIO-M1. The 10 µM concentration of GCV treatment showed nearly 50-60% cell viability in the all the CMV-TK transfected cell lines studied. The percentage of cell viability decreased with increasing concentration of GCV in Nthy-ori 3-1, Y79, and WERI-Rb1 cells (Figure 2A, 2C, 2D). In these cell lines, the cell viability was decreased under the EpCAM promoter regulated TK compared to that of the CMV promoter regulated TK in EpCAM^+ve^ cells. In MIO-MI cells, the cell viability rate is about 60% under the CMV promoter regulation of TK/GCV (Figure 2B) and 90% under the EpCAM promoter regulation of the gene. Furthermore, the cell viability significantly decreased under the EpCAM promoter regulated TK in Y79, WERI-Rb1, Nthy-ori 3-1 cells. These results suggest EGP2 promoter is expressing TK gene in EpCAM^+ve^ and not in the EpCAM^−ve^ cell lines.

### Presence of let-7 family miRNA in Retinoblastoma by real-time quantitative PCR

**Figure 3:**
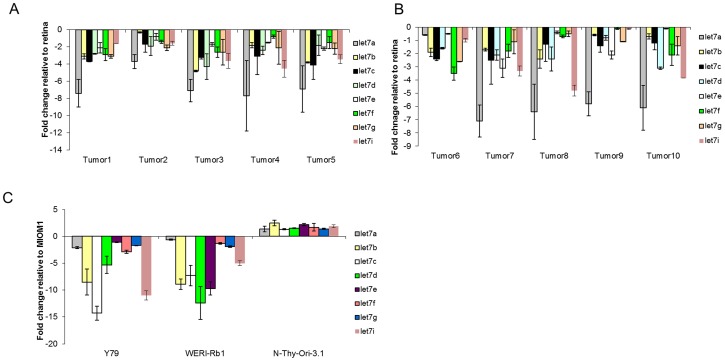
let-7 miRNA expression analysis in Retinoblastoma tumors and Retinoblastoma cell lines. The expression of let-7 miRNAs were quantified using real time PCR. The expression analysis of let-7 family miRNAs was evaluated in retinoblastoma primary tumors. The results were normalized against normal adult retina and were expressed in fold change values relative to normal adult retina (A, B). The expression analysis of let-7 family miRNAs was evaluated in retinoblastoma cell lines. The results were normalized against MIO-M1 and were expressed in fold change values relative to MIO-M1(C). miRNA fold change was calculated using 2^−▵▵CT^ method.

It has been reported that, let-7 family miRNAs are apoptotic, and highly conserved across species in sequence and function and deregulation of let-7 family leads to cancer [25]. We analysed the expression profile of let-7 family in (n = 10) primary retinoblastoma tumours and Y79, WER-Rb1, MIO-M1, Nthy-ori 3-1 cell lines (Figure 3). The let-7 family was down regulated in primary tumors when compared to that of the normal adult retina. let-7a was highly down regulated among the let- 7 family members in primary tumors (Figure 3A, B). let-7c and let-7d were highly down regulated in Y79 and WERI-Rb1 respectively, when compared to the MIO-M1cell line (Figure 3C). In Nthy-ori 3-1 cell line, all the miRNA were up regulated compared to MIO-M1 (Figure 3C). Furthermore, let-7b showed consistent down regulation in both the retinoblastoma cell lines when compared to other let-7 family members and showed high up-regulation in Nthy-ori 3-1. Hence, this (let-7b) was chosen to clone miRNA target sequences into our plasmid constructs.

### *In vitro* luciferase assay mediated by pCMV/EGP2-*luc* vectors

**Figure 4:**
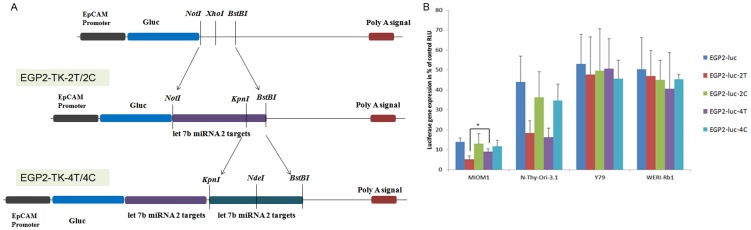
Schematic representations of EpCAM promoter with luciferase gene and miRNA targets vector constructs and expressional studies of the constructs using luciferase assay. miRNA target sequences two copies or four copies as detailed in Table 1. The reporter gene luciferase under CMV promoter was cloned in EpCAM-TK construct by removing TK gene and inserting luciferase gene. miRNA targets were cloned into 3′ UTR region of the expression vector containing Gluc gene under EpCAM promoter (A). The luciferase expression was quantified by secreted Gaussia luciferase. The plasmid constructs EGP2-luc, EGP2-luc-2T, EGP2-luc-4T, EGP2-luc-2C, and EGP2-luc-4C were transfected into MIO-M1, Nthy-ori 3-1, Y79, WERI-Rb1 cell lines. After incubation for 48 hrs the results were normalized to un-transfected cells and were expressed in relative light units (B). Luciferase levels are expressed as percentage mean ± S.D (n = 3). *p<0.05, **p<0.01, ***p<0.001 vs EGP-2-luc-2T.

The transfection of EGP2-TK and EGP2-luc constructs in EpCAM negative cell line showed significant expression of TK and luciferase genes (Figure1 C, E). A significant cytotoxicity upon GCV treatment observed in EpCAM negative cell line MIO-M1 upon EGP2-TK transfection with GCV treatment (Figure. 2B) could be due to leaky expression of EGP2 promoter in EpCAM negative cell lines. A similar leaky expression of the GFAP promoter was observed in normal astrocytes [34]. To regulate the non- specific expression of transgene driven by EGP2 promoter, we cloned let-7b targets at the 3’UTR region of EGP2-luc construct (Figure 4A). The cells were transfected with the EGP2-luc, EGP2-luc-2T EGP2-luc-4T, EGP2-luc-2C, EGP2-luc-4C plasmid constructs and the luciferase gene expression was analysed after 24 h. A significant reduction in the luciferase activity was observed with EGP2-luc-2T and EGP2-luc-4T when compared to EGP2-luc in MIO-M1 (EpCAM^–ve^/let-7b^up regulated^) and Nthy-ori 3-1 (EpCAM^+ve^/let-7b^up-regulated^) cells (Figure 4B). A further significant reduction in the luciferase activity in EGP2-luc-2T compared to EGP2-luc-4T was observed in MIO-M1 cells. However, in Y79 and WERI-Rb1 (EpCAM^+ve^/let7^down-regulated^) cells, we did not observe significant reduction in the luciferase activity of EGP2-luc-2T and EGP2-luc-4T compared to the EGP2-luc (Figure 4B). These results emphasises the need for the miRNA regulation of the transgene expression along with the tissue specific promoter for the targeted expression of transgene.

### Cell specific cytotoxicity by dual control vector transfection with GCV treatment was higher than ETK alone

**Figure 5:**
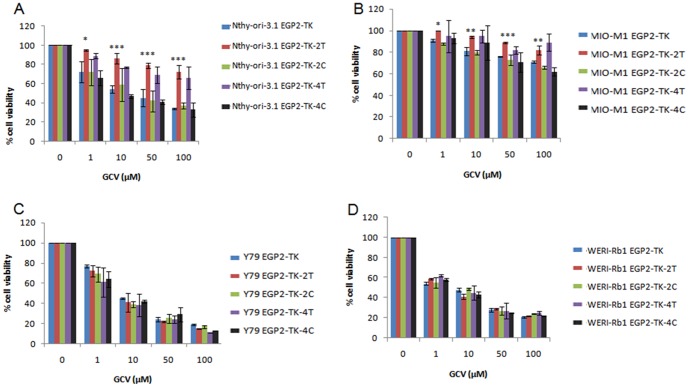
Targeted cell death of retinoblastoma cell lines regulated by miRNA targets. Cell viability assay by MTT was evaluated by transfecting the EGP2-TK, EGP2-TK-2T, EGP2-TK-4T, EGP2-TK-2C, EGP2-TK-4C plasmids into the cell lines, Nthy-ori-3-1 (A), MIO-M1 (B), Y79 (C), WERI-Rb1 (D). After 48 hrs of transfection, followed by GCV treatment at different concentrations (1, 10, 50, 100 µM) for 24 hrs, readings were measured at 570 nm using spectrophotometer. The results were normalized to untransfected cells. Cell viability was shown as percentage mean ± S.D (n = 3). *p<0.05, **p<0.01, ***p<0.001 vs. control.

We evaluated the targeted cytotoxicity induced by GCV treatment on EGP2-TK-2T and EGP2-TK-4T transfection compared to the controls EGP2-TK, EGP2-TK-2C and EGP2-TK-4C in all the 4 cell lines. The normal cell lines Nthy-ori 3-1 (Figure 5A) and MIO-M1 (Figure 5B) transfected with EGP2-TK-2T and EGP2-TK-4T constructs with GCV treatment resulted in significant lower cell death compared to the EGP2-TK. These cell lines showed significant less cytotoxicity mediated by EGP2-TK-2T compared to EGP2-TK-4T construct. In retinoblastoma cell lines Y79 (Figure 5C), WERI-Rb-1 (Figure 5D) transfected with EGP2-TK, EGP2-TK-2T, EGP2-TK-4T, EGP2-TK-2C and EGP2-TK-4C constructs, followed by GCV treatment showed no significant difference in cell death. These results show that cell specific cytotoxicity mediated by HSV-TK upon treatment with GCV drug was regulated by let-7b miRNA targets in the normal cell lines.

### EpCAM promoter and let-7b target sequence mediates expression of apoptotic marker in retinoblastoma cells

**Figure 6:**
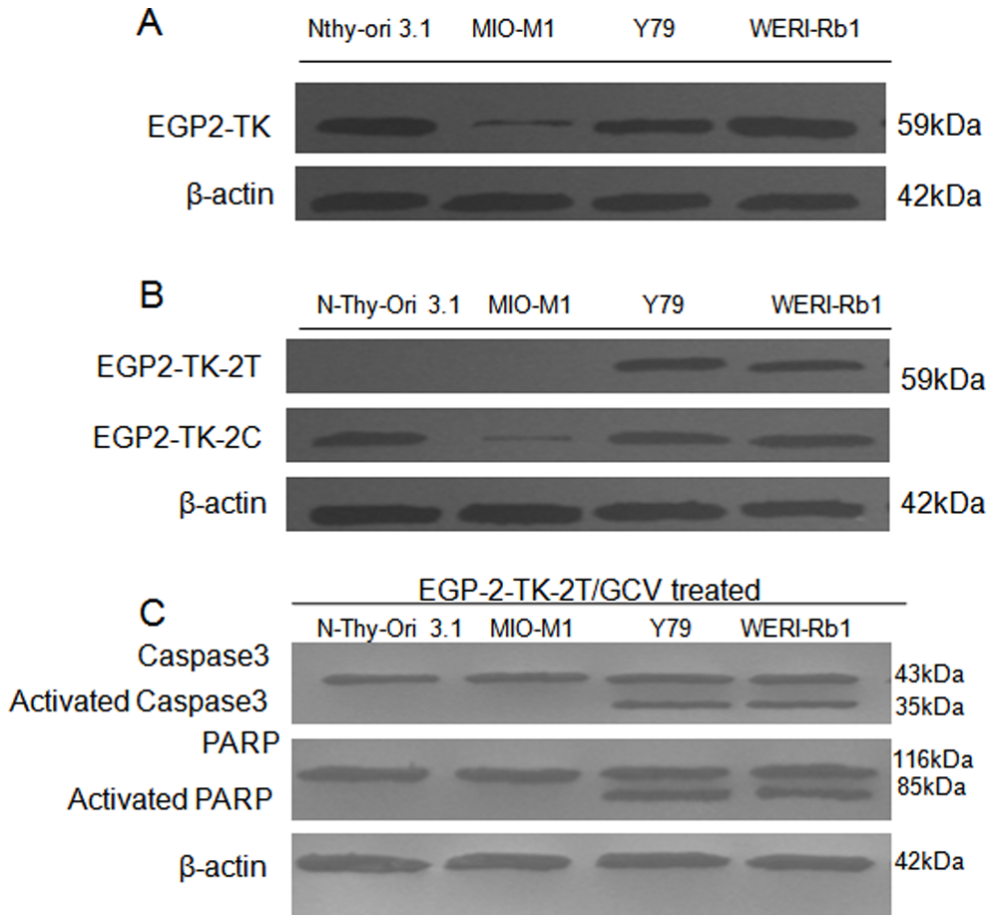
Targeted expression of TK protein and apoptotic regulation by EpCAM promoter and let-7b miRNA targets. Western blot analysis was performed for the presence of TK protein, EGP2-TK was transfected into Nthy-ori-3-1, MIO-M1, Y79 and WERI-Rb-1 cell lines (A). Western blot analysis was performed to assess the TK protein expression regulated under the influence of miRNA targets containing constructs, EGP2-TK-2T and EGP2-TK-2C were transfected into Nthy-ori-3-1, MIO-M1, Y79 and WERI-Rb-1 cell lines(B). To assess the apoptotic marker expression, transient transfection of,, EGP2-TK-2T, and GCV treatment for 48 hrs at 10 µM concentration in Nthy-ori-3-1, MIO-M1, Y79 and WERI-Rb-1cell lines was performed. Caspase and PARP expression was analysed by western blot. β-actin protein was used as loading control(C).

To control the reported leaky expression of the TK gene in the non-targeted normal cells (Figure 1 & 2), we transfected EGP2-TK, EGP2-TK-2T, EGP2-TK-2C constructs into the following cell lines: N-Thy-Ori-3.1, MIO-M1, Y79 and WERI-Rb-1. Since, we observed EGP2-TK-2T showed significant effect in controlling the transgene expression, we choose only EGP2-TK-2T for analysing apoptosis markers after GCV treatment. The EGP2-TK construct was highly expressed in EpCAM^+ve^ cell lines, Nthy-ori 3-1 (Figure 6A, lane 1), Y79 (Figure 6A, lane 3), WERI-Rb-1 (Figure 6A, lane 4), compared to EpCAM^−ve^ MIO-M1cell line (Figure 6A, lane 2). The transfection of EGP2-TK-2T construct restricted the TK expression only in retinoblastoma cell lines (Figure 6B, lane 3, 4) and not in normal cell lines such as MIO-M1 and Nthy-ori 3-1 even in the presence of EpCAM protein. The EGP2-TK-2T transfected retinoblastoma cell lines treated with GCV drug showed the apoptotic marker, activated Caspase3 and necrotic marker PARP-1 expression (Figure 6C, lanes 3, 4) but not in normal cell lines (Figure 6C, lanes 1, 2). To further validate the apoptotic markers expression, we performed immunofluroscence experiments where similar results were observed, which supports the apoptotic marker expression on western blot (figure S1). Instead of PARP we used Bcl-2 which is an anti apoptotic marker.

### EpCAM promoter and let-7b miRNA target sequence mediates expression of thymidine kinase and apoptotic marker in breast cancer cell lines

**Figure 7:**
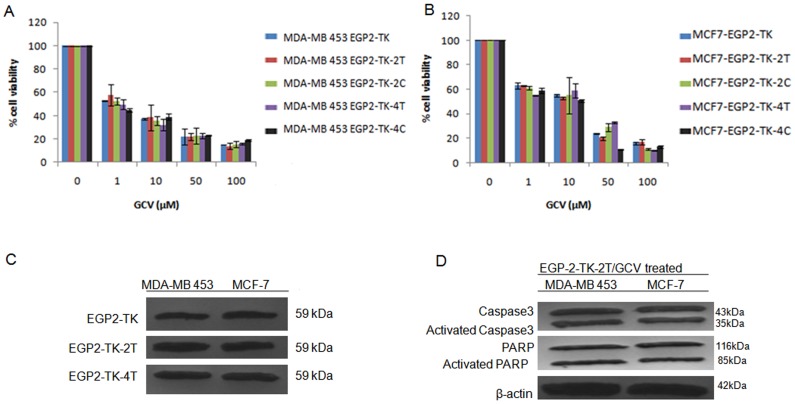
Cytotoxicity and apoptotic regulation by EpCAM promoter and let-7b miRNA targets in MDA-MB-453 and MCF7. Cell viability assay by MTT was evaluated by transfecting the EGP2-TK, EGP2-TK-2T, EGP2-TK-4T, EGP2-TK-2C, EGP2-TK-4C plasmids into the cell lines MDA-MB-453 (A) MCF7 (B). After 48 hrs of transfection, followed by GCV treatment at different concentrations (1, 10, 50, 100 µM) for 24 hrs. The results were normalized to un-transfected cells. Cell viability was shown as percentage mean ± S.D (n = 3). *p<0.05, **p<0.01, ***p<0.001 vs. control. The TK protein expression driven by EGP2 promoter and miRNA regulation was evaluated by transient transfection of EGP2-TK, EGP2-TK-2T, EGP2-TK-2C and treatment with GCV at 10 µM concentration for MDA-MB 453 and MCF7. After 48 hrs of transfection western blot analysis of TK protein expression normalized against the β-actin protein (C). Western blot analysis of apoptotic marker expression normalized against β-actin protein (D).

In order to know the efficacy of the dual control vector in other cancer cell lines we extended our strategy to MDA-MB-453 and MCF-7 breast cancer cell lines. Previous reports have showed strong expression of EpCAM in these cell lines [42]. However, the let-7 family profile status in these cell lines is still unknown. We evaluated the targeted cytotoxicity induced by GCV treatment on EGP2-TK-2T and EGP2-TK-4T transfection compared to the controls EGP2-TK, EGP2-TK-2C and EGP2-TK-4C in both the cell lines. We did not observe significant differences in the cytotoxicity mediated by TK suicide gene in MDA-MB 453 (Figure 7A) and MCF7 (Figure 7B) cells. Furthermore, presence of TK suicide protein in the western blot analysis confirms the activity of the EpCAM promoter and lack of let-7b miRNAs for transcriptional regulation of suicide gene in these cell lines (Figure7C).These results are in accordance with the results obtained using Y79 and WERI-Rb1 retinoblastoma cell lines. The expression of apoptotic marker, activated Caspase3, and necrotic marker PARP-1 confirms that these cells undergo apoptotic pathway (Figure 7D).

## Discussion

The potential use of HSV-TK/GCV suicide gene therapy in treatment of cancer has been well established [43], [44], [45], [46], [47]. However the suicide gene expression is universal in targeting non-cancerous normal tissues in addition to the cancer tissues. The non-specificity of the suicide gene expression in non-cancerous tissue is the major limitation for its clinical applications. The HSV-TK/GCV mediated toxicity in the normal cells has been addressed, by introducing the cancer tissue specific promoter to restrict the expression of suicide gene [18], [21], [22]. The activity of these promoters is restricted to the tissues where transcriptional activators are present. Even though it is a promising strategy, there are limitations as these promoters tend to show leaky expression in the non-cancerous cells such as MIO-M1 cell lines (Non-Neoplastic retinal glia) (present study). Here, we addressed a hypothesis that leaky promoter expression can be controlled by introducing miRNA target regulation to restrict the expression of the suicide gene to EpCAM positive cancer cells.

The EpCAM promoter was chosen as it has been regulated by the EpCAM protein which was shown earlier that it is over expressed in many primary tumors (Head & Neck cancer, Gastric cancer, Colorectal cancer) including retinoblastoma [23]. The EpCAM is over expressed in Y79 and WERI-Rb1 retinoblastoma cell lines [29] and Nthy-ori 3-1 normal cell line. MIO-M1 is a non-cancerous cell line derived from retinal Müller glia which shows no expression of EpCAM protein. However, the EpCAM promoter with luciferase reporter gene at the downstream of it was active in this cell line. This shows that the promoter is active in the cell line and is exhibiting a leaky expression. Furthermore, when we performed the cell viability assay with HSV-TK/GCV we observed that there is cell death in all the cell lines confirming the leaky expression of the EpCAM promoter in the MIO-M1 cells (EpCAM^−ve^). To regulate the leaky EpCAM promoter expression in the normal tissue we employed the strategy of using miRNA targets that are cancer tissue specific at the downstream of the HSV-TK suicide gene. Earlier Suzuki *et al*. (2008) and Brown et al (2007) have reported the use of hsa-miR-31, hsa-miR-127, and hsa-miR-143 miRNA targets to control the expression of the HSV-TK gene to glioblastoma cell lines.

The let-7 miRNA family is known to be down regulated in many cancers [25]. The expression of let-7 miRNA family members has not been reported in retinoblastoma primary tumors and cell lines. Though let-7b, let7d down regulation was reported in retinoblastoma [48], [49], we have profiled the let-7 family miRNAs in the present study. We observed that all let-7 family miRNAs were down regulated in the primary retinoblastoma tumors when compared to the normal adult retina. Nthy-ori 3-1 a normal thyroid cell line with over expression of EpCAM also exhibited higher expression of let-7 family miRNAs compared to MIO-M1 cell line. Furthermore, let-7b miRNA was highly up regulated in the Nthy-ori 3-1 cell line among the let-7 family members. Therefore, in our study we used let-7b miRNAs targets in the 3’UTR region of the EpCAM promoter driven suicide gene to restrict its expression to the cancer cells compared to the normal cells. The primary tumors are heterogeneous in nature whereas cell lines are homogenous in nature [34]. Therefore, we conducted further experiments in the cell lines.

**Figure 8:**
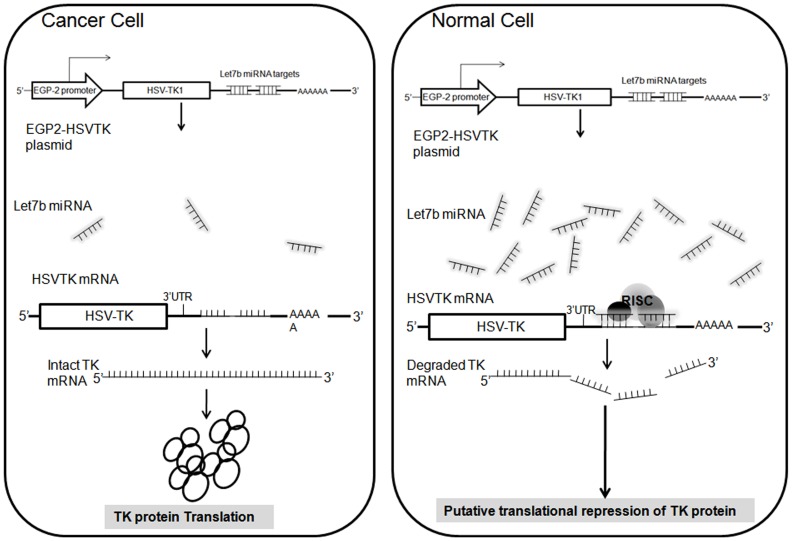
Schematic representation of EpCAM promoter mediated HSV-TK suicide gene expression regulated by let-7 miRNA targets. In cancer cells EpCAM promoter mediated suicide gene expression with miRNA targets was high due to presence of cell specific expression of the promoter and lack of sufficient let-7 miRNA copies. Where as in Normal cells due to the presence of high copy number of let-7 miRNAs complementation to its target sequences which recruits RISC factor and degrades the mRNA resulting in “putative” translational repression of TK protein.

We designed the let-7b miRNAs targets to be perfect complement to the let-7b miRNAs as this likely maximizes the binding efficiency and ensure the transcript cleavage. The let-7b miRNA targets were cloned at the 3’UTRs of the HSV-TK and luciferase genes. Upon transfection of the plasmid constructs we observed a significant reduction in the expression of the luciferase gene with the EGP2-luc-2T, EGP2-luc-4T constructs when compared to the luciferase expression with EGP2-luc. The reduction in luciferase expression in the MIO-MI and Nthy-ori 3-1 cells is due to the translational repression of the luciferase enzyme by the complementation between the miRNAs and its targets. The luciferase with EGP2-luc-2T showed significant reduction compared to EGP2-luc-4T in the MIO-M1 cell line. The reduction in the luciferase expression could be due to the “sponge” effect. In such instances, increasing the number of targets are reported to guide multiple RISC factors to the single transcript and sequester the complementary miRNAs and its targets thus limiting the mRNA cleavage [50]. The differences in the luciferase expression with EGP2-luc-2T and EGP2-luc-4T in MIO-M1 and Nthy-ori 3-1cell lines could be cell specific as there is no consensus in the number of targets which proves efficacious for miRNA regulated transgene expression [51]. In Y79 and WERI-Rb1, MDA-MB-453, MCF-7 cell lines, we did not observe any significant reduction in the luciferase expression. This may be due to the lack of let-7b miRNAs to transcriptionally regulate the luciferase gene. HSV-TK/GCV treatment involves inhibition of the DNA synthesis by phosphorylated GCV and interfere in replication process which damages both nuclear and mitochondrial DNA [50]. In agreement with the luciferase expression, reduced cytotoxicity was observed in EGP2-TK-2T and EGP2-TK-4T in the normal MIO-M1 (EpCAM^−ve^/let-7^up-regulated^) and Nthy-ori 3-1 (EpCAM^+ve^/let-7 ^up-regulated^) cell lines. In Y79, WERI-Rb1, MDA-MB-453, MCF-7 cell lines a significant difference was not observed in the cytotoxicity with and without let-7b target sequences. The cause of cell death in the cancer cells was further investigated using apoptotic and necrotic factors. We observed the activation of apoptosis and necrotic markers in MDA-MB-453, MCF-7, Y79 and WERI-Rb1 retinoblastoma cells but not in MIO-M1 and Nthy-ori 3-1normal cell lines. The let-7b miRNA based regulation reduced the suicide gene expression in the normal cells resulting in non- activation of apoptosis and necrosis. These results show that transgene expression can be regulated by both cell specific promoter and levels of miRNA (Figure 8).

In summary, the present study provides evidence that incorporating miRNA regulation into a tissue specific vector can provide an important additional control over the transgene expression. This expression system reduces the off target expression of the suicide gene in the normal cells, reducing the side effects associated with the cancer gene therapy. This dual regulation system for transgene involving EpCAM and let-7 miRNA can be used, not only in retinoblastoma and breast cancer cell lines but any EpCAM positive and let-7 negative cancer cells.

## Supporting Information

Figure S1 **Immunofluorescence analysis of reduced TK leaky expression by let7b miRNA targets.** The detection of apoptotic marker, cleaved Caspase 3 and anti-apototic marker Bcl-2 were analysed by immunofluorescence after transfection of the EGP2-TK, EGP2-TK-2T, EGP2-TK-2C, plasmids into the cell lines, MIO-M1 (A), Nthy-ori-3.1 (B), WERI-Rb1 (C), Y79 (D). After 48 h of transfection, followed by GCV treatment at 10 µM concentration for 24 h cells were fixed and stained for cleaved Caspase 3 (Green) and Bcl-2 (Red) marker expression along with DAPI (Blue) staining to identify nuclear morphology. EGP-2-TK, EGP-2-TK-2T, EGP-2-TK-2C transfected and GCV treated cell lines N-Thy-Ori-3.1, MIO-M1, Y79, WERI-Rb-1 were stained with markers for the apoptosis (cleaved Caspase3, Green staining) and anti-apoptosis (Bcl-2, Red staining). We found that transfection of EGP-2-TK showed a mild positivity for both apoptotic and anti-apoptotic markers which could be the result of TK leaky expression in MIO-M1 cell line (A), whereas transfection of EGP2-TK-2T increases the anti-apoptotic marker Bcl-2 staining and transfection of EGP2-TK-2C reverses the effect of EGP2-TK-2T. In Nthy-Ori-3-1 cell line (B), transfections with EGP2-TK resulted in strong staining for Caspase 3 and in EGP2-TK-2T transfection strong Bcl-2 staining were seen relative to very mild Caspase 3 staining. Weri-Rb-1 (C) and Y79 (D) cell lines showed strong staining for Caspase 3 with very minimal staining for Bcl-2 in all the transfections.
(TIF)

Table S1 Clinicopathological details of retinoblastoma samples used in this study.
(DOC)
