# Supplementary figures and images for: Targeted Expression of Suicide Gene by Tissue-Specific Promoter and MicroRNA Regulation for Cancer Gene Therapy
Source: PLoS One. 2013 Dec 31;8(12):e83398. doi: 10.1371/journal.pone.0083398 (PMC3877029; doi:10.1371/journal.pone.0083398)

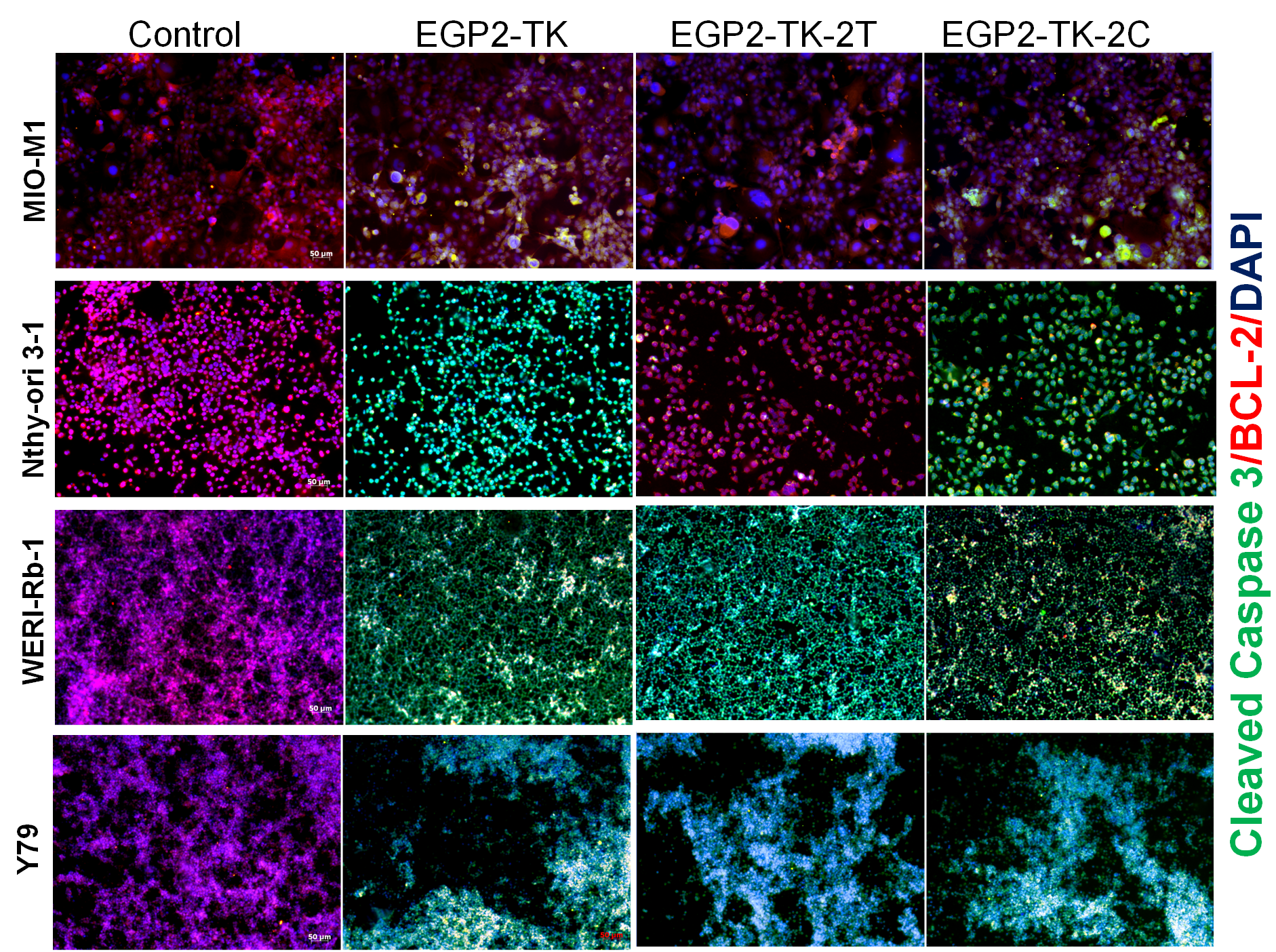

Supplement: Figure S1 — Immunofluorescence analysis of reduced TK leaky expression by let7b miRNA targets. The detection of apoptotic marker, cleaved Caspase 3 and anti-apototic marker Bcl-2 were analysed by immunofluorescence after transfection of the EGP2-TK, EGP2-TK-2T, EGP2-TK-2C, plasmids into the cell lines, MIO-M1 (A), Nthy-ori-3.1 (B), WERI-Rb1 (C), Y79 (D). After 48 h of transfection, followed by GCV treatment at 10 µM concentration for 24 h cells were fixed and stained for cleaved Caspase 3 (Green) and Bcl-2 (Red) marker expression along with DAPI (Blue) staining to identify nuclear morphology. EGP-2-TK, EGP-2-TK-2T, EGP-2-TK-2C transfected and GCV treated cell lines N-Thy-Ori-3.1, MIO-M1, Y79, WERI-Rb-1 were stained with markers for the apoptosis (cleaved Caspase3, Green staining) and anti-apoptosis (Bcl-2, Red staining). We found that transfection of EGP-2-TK showed a mild positivity for both apoptotic and anti-apoptotic markers which could be the result of TK leaky expression in MIO-M1 cell line (A), whereas transfection of EGP2-TK-2T increases the anti-apoptotic marker Bcl-2 staining and transfection of EGP2-TK-2C reverses the effect of EGP2-TK-2T. In Nthy-Ori-3-1 cell line (B), transfections with EGP2-TK resulted in strong staining for Caspase 3 and in EGP2-TK-2T transfection strong Bcl-2 staining were seen relative to very mild Caspase 3 staining. Weri-Rb-1 (C) and Y79 (D) cell lines showed strong staining for Caspase 3 with very minimal staining for Bcl-2 in all the transfections. (TIF) [file pone.0083398.s001.tif]
