# Supplementary material for: Targeted Expression of Suicide Gene by Tissue-Specific Promoter and MicroRNA Regulation for Cancer Gene Therapy
Source: PLoS One. 2013 Dec 31;8(12):e83398. doi: 10.1371/journal.pone.0083398 (PMC3877029; doi:10.1371/journal.pone.0083398)
Supplement: Table S1 — Clinicopathological details of retinoblastoma samples used in this study. (DOC) [file pone.0083398.s002.doc]

**Table S1:** Clinicopathological details of Retinoblastoma samples used in this study

| S. NO | AGE | SEX | LATERIALTY | HISTOPATHOLOGICAL INFORMATION |
| --- | --- | --- | --- | --- |
| 1 | 4 years | F | unilateral | Poorly Differentiated, Focal choroidal invasion measuring >3mm .No invasion in optic nerve and surgical end. Tumour cells are invading anterior fibres of sclera |
| 2 | 2 years | M | unilateral | Poorly Differentiated, No invasion of Choroid Pre Laminar Invasion .No invasion in Post-Laminar and Surgical End of Optic Nerve |
| 3 | 4 years | F | unilateral | Poorly Differentiated, Multiple foci of retinal pigment epithelium Invasion, Multiple Focal of Choroidal Invasion measuring >3mm. Prelaminar and Minimal Laminar Invasion of Optic Nerve. No Invasion into Post-Laminar portion and Surgical End of Optic Nerve. |
| 4 | 7 days | F | Bilateral | Well Differentiated, Focal Choroidal Invasion measuring >3mm. Invasion in Pre Laminar, Minimal Laminar of optic nerve. No invasion in Post-Laminar and Surgical End of Optic Nerve. |
| 5 | 4 years | M | Bilateral | Moderately Differentiated, tumour adherent to retinal pigment epithelium focal invasion of choroid measuring <3mm. Invasion in prelaminar of optic nerve .No invasion in laminar , post laminar portion and surgical end of optic nerve |
| 6 | 5 years | F | unilateral | well differentiated, extensive invasion of retinal pigment epithelium ,choroidal invasion measuring >3mm . Invasion in prelaminar and no invasion in laminar ,post laminar and surgical end of optic nerve |
| 7 | 2 ½ months | M | unilateral | poorly differentiated, Invasion of the iris stromal present. Choroidal invasion measuring >3mm. no invasion of the optic nerve |
| 8 | 7 months | F | unilateral | Well Differentiated, Choroidal Invasion measuring <3mm, Invasion in Pre-Laminar, Laminar and Post-Laminar of Optic Nerve .No invasion in Surgical End of Optic Nerve . |
| 9 | 3 years | F | unilateral | Moderately differentiated. choroidal invasion >3mm.Invasion in prelaminar and no invasion in laminar , post laminar portion and surgical end of optic nerve is free from tumour |
| 10 | 10 months | M | unilateral | Moderately differentiated, No invasion in choroidal ,no invasion in optic nerve. surgical end . |
